# Supplementary material for: Enhancing genome recovery across metagenomic samples using MAGmax
Source: Bioinformatics. 2025 Sep 26;41(10):btaf538. doi: 10.1093/bioinformatics/btaf538 (PMC12534905; doi:10.1093/bioinformatics/btaf538)
Supplement: btaf538_Supplementary_Data [file btaf538_supplementary_data.zip › Supplementary_Materials.pdf]

Supplementary materials for enhancing genome recovery across metagenomic samples using MAGmax

Arangasamy Yazhini[1*] and Johannes Söding[1,2,3,*]

[1]Quantitative and Computational Biology, Max-Planck Institute for Multidisciplinary Sciences, Göttingen, Germany
[2]International Max-Planck Research School for Genome Sciences, University of Göttingen, Germany.
[3]Campus Institute Data Science (CIDAS), University of Göttingen, Germany.
[*]Correspondence: yazhini@mpinat.mpg.de and soeding@mpinat.mpg.de

Supplementary Tables

Table 1: The number of bin pairs with ≥99% ANI in the dRep output

Binner	Dataset	Contamination level (%)	No. of pairs
VAMB	Human gut Honduras	5	1
MetaBAT2	Human gut Honduras	5	1
GenomeFace	Human gut Honduras	5	2
VAMB	Neonatal gut	5	2
GenomeFace	Neonatal gut	5	6
VAMB	Human gut Honduras	10	1
MetaBAT2	Human gut Honduras	10	1
GenomeFace	Human gut Honduras	10	2
VAMB	Black soil	10	1
VAMB	Neonatal gut	10	3
GenomeFace	Neonatal gut	10	4

Supplementary Figures

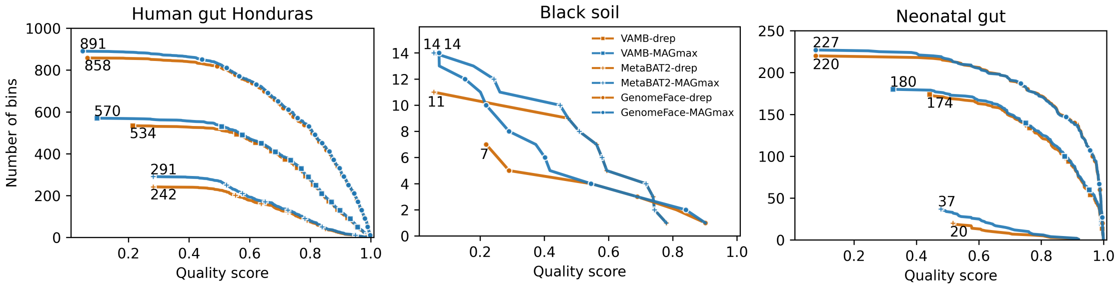

Figure 1: Complementary cumulative frequency of dereplicated bins as a function of quality score at 10% contamination.

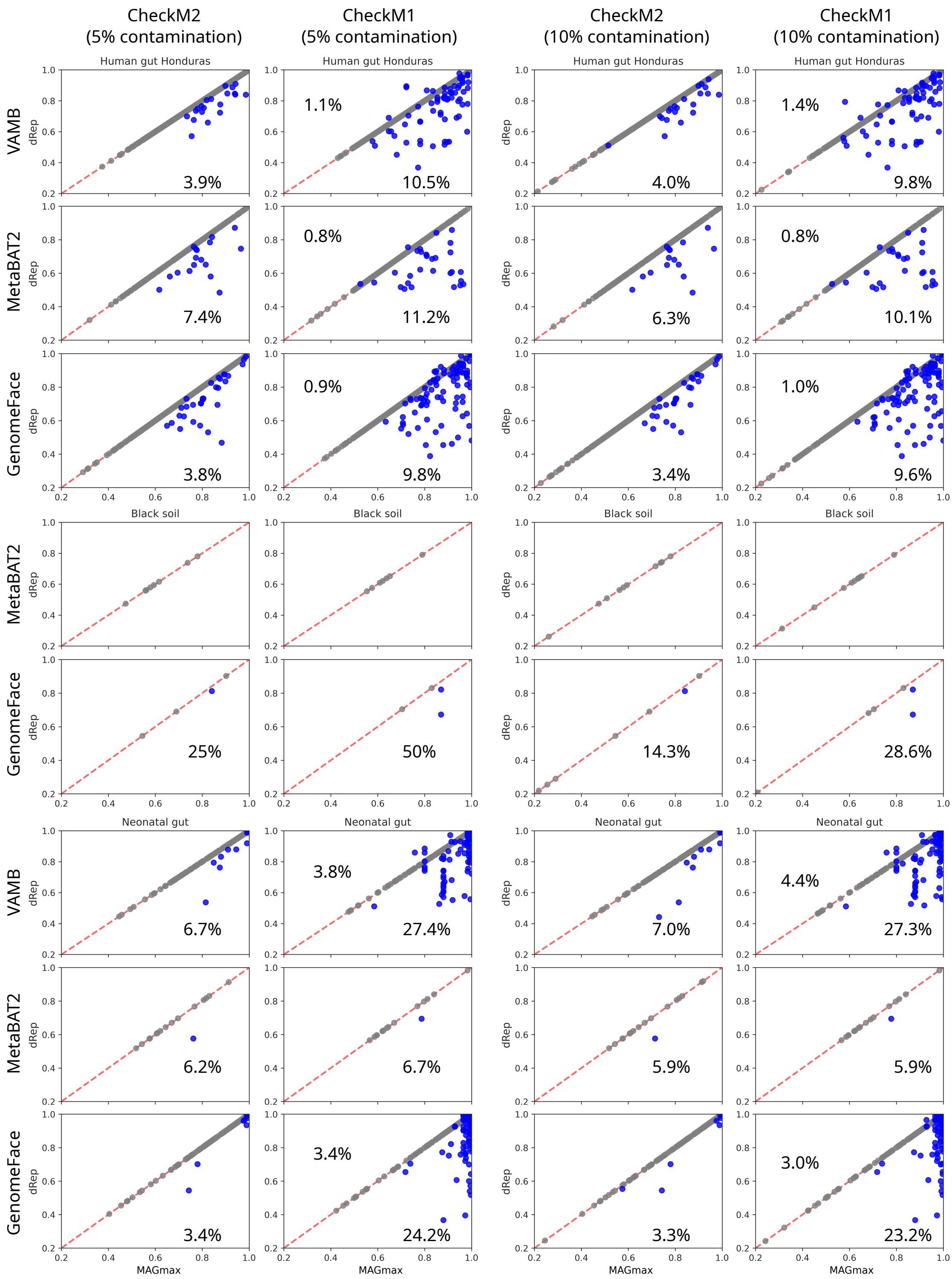

Figure 2: Comparison of quality scores of dRep bins and their corresponding MAGmax bins based on genomic cluster membership using CheckM2 (left) and CheckM1 (right) prediction. Numbers in percentage indicate the fraction of bins that differ and have a higher quality score than their counterpart in the other tool.

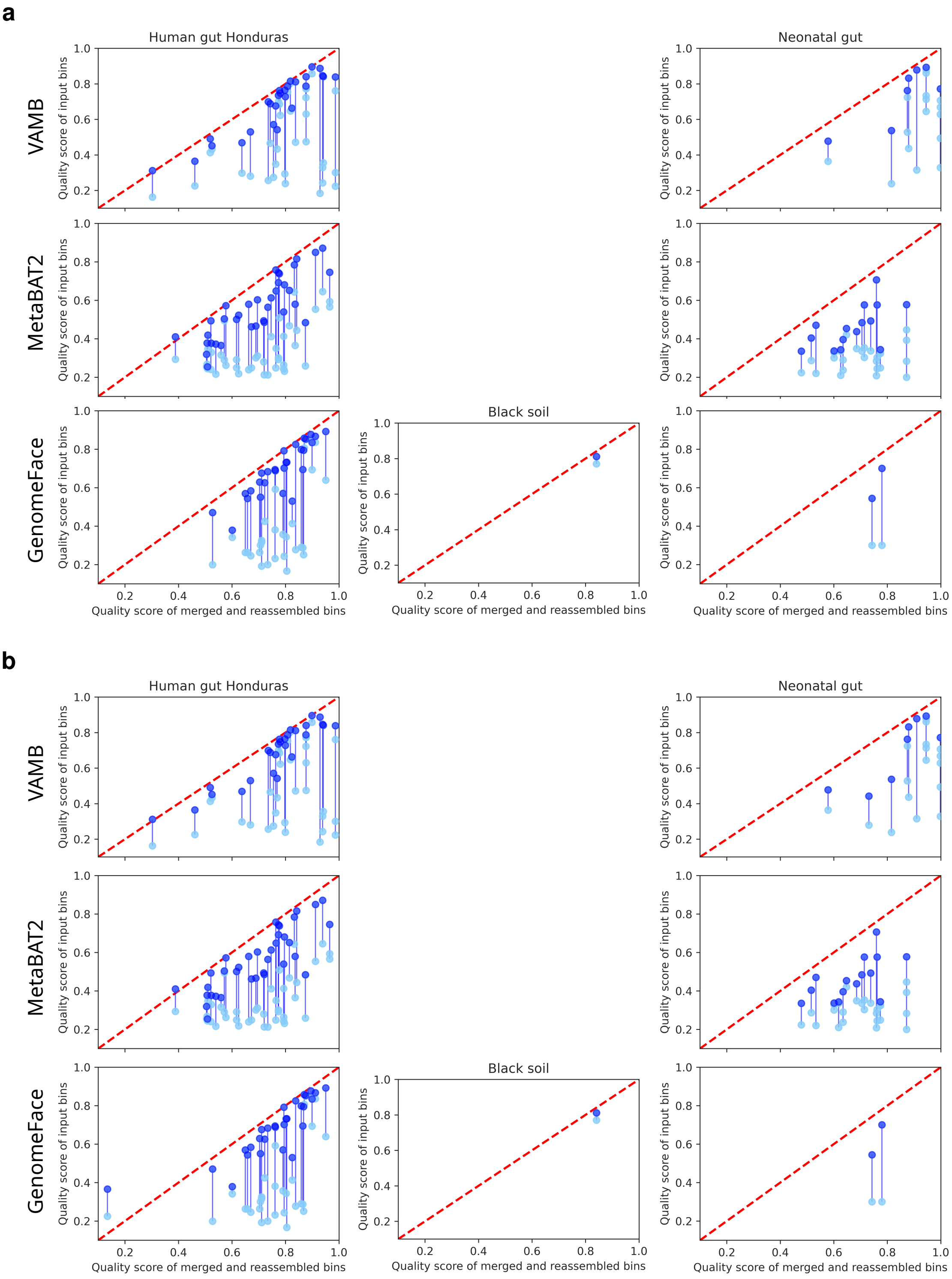

Figure 3: Comparison of quality scores between input bins (y-axis) and their corresponding merged and reassembled bins (x-axis) at 5% contamination (a) and 10% contamination (b). Blue circles represent the highest-quality bins within each genomic cluster, while cyan circles indicate the other input bins. Vertical lines connect the input bins that were used for merging and reassembly.
